# P02-16 Implementation of a program based on adapted physical activity and recommendations for second cancers prevention for adolescents and young adults with cancer: PREVAPAJA study

**DOI:** 10.1093/eurpub/ckac095.035

**Published:** 2022-08-29

**Authors:** Rodolf Mongondry, Olivia Perol, Perrine Marec-Berard, Lidia Delrieu, Olivia Febvey-Combes, Axel Lion, Serge Marvalin, Nora Moumjid-Ferdjaoui, Béatrice Fervers, Helen Boyle, Julien Carretier

**Affiliations:** 1 Prévention Cancer Environnement, Centre Léon Bérard, Lyon, France; 2 AYAs Department- Treatment of AYA’s Pain Unit, Centre Léon Bérard, Lyon, France; 3 HESPER- Health Services and Performance Research, University Claude Bernard Lyon 1, Lyon, France; 4 AYAs Department- Treatment of AYA’s Pain Unit, Institute of Hematology and Oncology Pediatrics, Lyon, France

**Keywords:** Adolescents and Young adults, Cancer, Physical Activity

## Abstract

**Background/Objectives:**

About 1,000,000 new cases of cancer in Adolescent and Young Adults (AYAs) are diagnosed annually worldwide. . While their long term survival is about 80%, they are six times more likely to develop a second primary cancer (SPC) compared to their peers. This risk is multifactorial and depends on the type of first cancer, treatment received and prevalence of risk factors. PREVAPAJA aimed to implement a clinical program based on physical activity (PA) and cancer prevention recommendations for AYAs with cancer at Centre Léon Bérard-AYAs Department.

**Methods:**

The study was conducted at Leon Berard Comprehensive Cancer Centre among patients aged 15-25 years. AYAs attended PA sessions during the active treatment period and were individually informed on SPC risk prevention. PA, sedentary, anthropometrics, quality of life and fatigue were assessed at baseline (T1) and at the end of treatment (T2). PA level and intention of changes in health behaviors were assessed by phone 1 year after T1.

**Results:**

68 AYAs (median age=19 years) were enrolled in 2016-2017). The results showed an improvement in PA level during and at distance of the intervention, with also a reduction of sitting time. Fatigue decreased between T1 and T2 (*p*>0.003) and overall quality of life improved significantly between T1 and T2 (*p*>0.001).

**Conclusions:**

This study showed the feasibility of implementing a clinical program based on PA intervention and cancer prevention recommendations for AYAs with cancer. It responded to AYAs' needs for support and discussions regarding PA recommendations and ways to prevent SPC. Beneficial outcomes of this program should encourage to systematically proposing PA intervention in combination with information exchanges with AYAs with cancer.

